# How composition of device-measured 24-h movement behaviors differs between office and remote workdays among hybrid workers?

**DOI:** 10.1186/s12966-026-01959-0

**Published:** 2026-07-28

**Authors:** Kristin Suorsa, Miika Tuominen, Sanna Pasanen, Jesse Pasanen, Nidhi Gupta, Sari Stenholm, Tuija Leskinen

**Affiliations:** 1 Department of Public Health, University of Turku and Turku University Hospital, Turku, Finland; 2 Centre for Population Health Research, University of Turku and Turku University Hospital, Turku, Finland; 3 Department of Musculoskeletal Disorders and Physical Work Load, National Research Centre for the Working Environment, Copenhagen, Denmark; 4 Research Services, Turku University Hospital and University of Turku, Turku, Finland

**Keywords:** Time-use, Work, Remote work, Physical activity, Sedentary behavior, Sleep, Compositional data analysis, Occupational health

## Abstract

**Background:**

There is limited evidence, especially from the post‑pandemic period, on how remote workdays differ from office workdays in 24‑h movement behaviors, that is, sleep, sedentary behavior (SED), light physical activity (LPA), and moderate-to-vigorous physical activity (MVPA), and how time is reallocated when work shifts from the office to remote environment. This study examined differences in 24-h movement behaviors between office and remote workdays among hybrid workers.

**Methods:**

Overall, 97 university employees (84% women, mean age 42 ± 10 years) self-reported their time in bed (TiB) and wore a thigh-worn Fibion SENS sensor for 7 consecutive days, including at least one office and one remote workday (on average 2.4 office and 2.6 remote workdays). Sensor-identified body postures and activity types were further categorized into SED, LPA, and MVPA. The compositional linear mixed models were used to examine within-individual differences in 24-h movement behaviors between remote and office workdays, adjusting for sex, age, and body mass index (BMI). To aid further interpretation, time flow analysis was used to examine how time was redistributed between movement behaviors when changing the work environment from office to remote.

**Results:**

The ratio of active behaviors (LPA, MVPA) to passive behaviors (TiB, SED) was lower on a remote workday compared to office workday (*p*<.0001). This corresponded to 14 min more TiB, 44 min more SED, 37 min less LPA, and 21 min less MVPA (compared with office workday including 7.6 h TiB, 10.6 h SED, 5.0 h LPA, and 49 min MVPA). Moreover, the ratio of LPA to MVPA was higher on a remote workday when compared to an office workday (*p*<.0001). No significant differences were observed in the ratio of SED to TiB (*p* = .15). Mainly, the time spent standing and cycling was replaced with SED and TiB when changing the work environment from office to a remote.

**Conclusions:**

Compared with office workdays, remote workdays included slightly longer TiB and substantially more SED, alongside lower physical activity. In particular, the higher SED and lower physical activity observed during remote work suggest that exploring feasible ways to compensate for these behavioral differences may be warranted.

**Supplementary Information:**

The online version contains supplementary material available at https://doi.org/10.1186/s12966-026-01959-0.

## Background

The prevalence of remote work increased markedly during the COVID-19 pandemic and has remained at a high level, as statistics from 2023 to 2025 indicated that over one-fifth worked at least partly remotely in Finland, as well as in other European countries and in the US [1–3]. Remote work can affect daily sleep/time in bed (TiB), sedentary behavior (SED), light physical activity (LPA), and moderate-to-vigorous physical activity (MVPA), often referred to as ’24-h movement behaviors’ [4, 5], due to reduced commuting and greater flexibility in scheduling working hours, tasks, and breaks during the workday.

Evidence, largely from studies conducted during the COVID-19 pandemic, suggests that remote work is associated with more SED and less physical activity [6]. Comparing remote workers to office workers has further shown that remote workers tend to have longer device-based sleep [7]. Moreover, studies comparing remote and office workdays within the same individuals, which control for time-invariant confounders as well as reduce selection bias, have similarly shown longer self-reported TiB [8–10] and device-based sleep [11]. For device-based wake-time movement behaviors, these studies also report higher daily SED [8, 12–14], lower total physical activity [12, 13], and lower daily MVPA [14, 15] on remote versus office workdays. In addition, higher worktime SED and lower worktime physical activity have been observed during remote workdays [12, 13, 16]. More recent findings in the WORKDAY study also indicated that remote workdays included more device-based SED, less LPA, and MVPA compared to office workdays [17].

However, existing research has several important limitations. First, most prior studies have disregarded TiB as an important component of a 24-h day [12–16, 18]. Second, wake-time movement behaviors have been examined in isolation from each other [10–12, 15, 16], which does not take into account the fact that time spent in movement behaviors from a 24-h composition, with any change in one behavior resulting in a corresponding increase or decrease in another [19, 20]. Third, those rare studies examining within-individual differences in the composition of all 24-h movement behaviors between remote and office workdays [8, 9] have been limited to the COVID-19 pandemic time. These studies among middle-aged office workers in Sweden found longer TiB relative to time awake on a remote workday when compared to the office workday [8, 9], and either no differences in wake-time behaviors [9] or slightly higher SED relative to time spent awake [8]. Factors other than remote work itself may have influenced these findings, such as pandemic-related restrictions, social isolation, and the abrupt shift to remote work, often without adequate home-office equipment or established remote-working practices [6, 21]. Fourth, to the best of our knowledge, no prior studies have examined in detail how time spent in different movement behaviors is redistributed when changing the work environment from office to remote. Consequently, it remains unclear how remote workdays differ from office workdays in the 24-h composition of daily behaviors under post-pandemic working conditions.

To fill these gaps in previous studies, we measured 24-h movement behaviors with devices among hybrid workers during post-pandemic times. Our aim was to examine within-individual differences in 24-h movement behaviors between office and remote workdays. We applied compositional data analysis (CoDA), which addresses the codependency between movement behaviors [19, 20, 22], and time flow analysis, which allows for modelling how time spent in movement behaviors is redistributed when changing the work environment from office to remote. Understanding these behavioral shifts helps to identify potential targets for future intervention studies aiming to promote healthier daily activity patterns in remote work environments.

## Methods

### Study population

The daily physical activity among hybrid workers (WORKDAY study) was set to compare daily physical activity and sedentary behavior between office and remote workdays [17]. Eligible participants were those employees who were office workers and able to do their work also remotely. Study participants were recruited from the campus area of the University of Turku, the Turku University hospital, and the Turku University of Applied Sciences, Finland, by advertising the study in the internal bulletins, units’ email lists, and campus buildings’ noticeboards. Detailed information on the recruitment procedure is described elsewhere [17]. Overall, 108 persons answered the registration questionnaire, of which four responses were direct refusals, with five further participants eventually dropping out due to scheduling issues. Of those who agreed to participate, 99 (84% women) participants were available for the measurements, wore the sensor, and responded to the study questionnaire. Two participants were excluded due to having less than one valid (≥ 10 h sensor wear time during self-reported waking hours) office workday and one valid remote workday, leading to an analytical sample of 97 participants. All the included participants wore the sensor for at least 6 days, and all but three participants completed the full 7-day measurement period.

The WORKDAY study was approved by the Ethics Committee for human sciences at the University of Turku (13/2023). All participants gave their written informed consent before the measurements.

### Assessment of 24-h movement behaviors

Physical activity and SED were measured with the Fibion SENS Motion System, which includes a triaxial SENS motion^®^ sensor, a smartphone application, and a web-based application [23]. Detailed measurement protocol is reported elsewhere [17]. After initialization of the sensor (to 12,5 Hz sampling frequency), sensors were given to participants and attached with an adhesive waterproof film directly to the skin on the mid-thigh. The participants were asked to wear the sensor continuously for 24 h per day for seven consecutive days, including during water-based activities, but to remove it for sauna or in case of skin irritation. The measurement period was requested, but not restricted, to include two office workdays and two remote workdays. The participants were also instructed to fill in a daily log during their measurement period to record daily work environments (office, remote, combined), daily working hours, bedtimes, and wake times. Measurements were conducted during all seasons of the year (winter 10%, spring 43%, summer 14%, and autumn 32%).

Accelerometry data were sampled in 5-s epochs and automatically categorized into time spent in the predefined activity categories using a validated built-in algorithm [23, 24]. The activity categories included: lying or sitting awake as SED; standing, sporadic walking, and continuous light walking as LPA; cycling, moderate intensity activity (e.g., brisk walking), and vigorous intensity activity (e.g., running) as MVPA. Sporadic and continuous light walking were distinguished from moderate-intensity activity using an activity intensity cut-off of 50, defined as the average magnitude of movement derived from high-pass filtered tri-axial accelerometer data [24]. TiB was estimated from daily logs as the duration between participants’ self-reported time of going to bed and time of waking up. Non-wear time of the sensor was defined as periods when activity categorization was not possible and excluded using the predefined “no data” category [23].

A measurement day was defined as a period from midnight to midnight, and a valid measurement day was defined as a measurement day with at least 10 h of sensor wear time during self-reported waking hours. Workdays including both office and remote worktimes were excluded from the analysis (*n* = 19 days, 4% of all valid workdays). Daily logs were used to classify office and remote workdays, and to distinguish between worktime and non-worktime. The weekly means of waking behaviors were calculated for valid days separately for each day type. The weekly mean of TiB was calculated from all valid measurement days including self-reported bedtimes and wake times. Due to the midnight-to-midnight definition of the measurement day, TiB consisted of two periods: [1] from midnight to the self-reported wake time, and [2] from the self-reported bedtime to midnight.

### Participant characteristics

Age, sex, occupational title, self-rated health, self-reported height and weight, current smoking status, and commuting mode were obtained using a web-based study questionnaire. Occupational titles were coded according to the ISCO classification [25] into professionals (ISCO 1–2) or associate professionals/service workers (ISCO 3 − 5). Self-rated health was assessed by a 5-point scale: good, rather good, average, rather poor, poor [26]. Body Mass Index (BMI) was calculated from self-reported body weight and height as kg/m^2^ and categorized into normal weight (< 25.0 kg/m^2^), overweight (25 to < 30 kg/m^2^), and obese (≥ 30 kg/m^2^) [27]. Based on the most common commuting mode reported by the participants, they were categorized into (1) passive commuters, i.e., those commuting by passive modes (car, public transport), and (2) active commuters, i.e., those commuting by active modes (walking, cycling).

### Statistical analysis

Descriptive information on participant characteristics is presented using means and standard deviations for continuous variables and frequencies and percentages for categorical variables.

### Compositional data analysis

The proportion of time spent in each behavior was treated as compositional data. The analyses were conducted using the packages *compositions* [28], *robCompositions* [29] and *nlme* [30] in R version 4.3.1 [31].

The dataset included six zero values for worktime MVPA, and because zeros cannot be included in CoDA, these values were imputed close to zero using the R-package *robCompositions*. An isometric logratio (ilr) transformation was used to map the compositional data into real-valued coordinates, which reduces the dimensionality of the data and allows standard statistical methods to be used [22]. The specific type of ilr coordinates used in this study were *balance coordinates* [32]. The balance coordinates were formed using sequential binary partitioning. For workdays, first, active behaviors, that is, a combination of LPA and MVPA, were selected as positive (placed in the numerator of the balance coordinate 1), and the passive behaviors, that is, a combination of TiB and SED, were selected as negative (placed in the denominator of the balance coordinate 1). The positive values of this coordinate indicate that the relative contribution of active behaviors exceeds that of passive behaviors, and vice versa. For the second balance coordinate, LPA was selected as positive and MVPA as negative. Finally, for the third balance coordinate, SED was selected as positive and TiB as negative. For workdays’ worktime and non-worktime, separately, balance coordinate 1 was formed by selecting SED as positive and a combination of LPA and MVPA as negative, while balance coordinate 2 was formed by selecting LPA as positive and MVPA as negative.

After the coordinate transformation was applied to the compositional data, linear mixed models were fitted for each of the coordinates (workdays: (1) active vs. passive behaviors, (2) LPA vs. MVPA, and (3) SED vs. TiB; worktime/non-worktime: (1) SED vs. LPA and MVPA, and (2) LPA vs. MVPA) to compare the composition of 24-h movement behaviors between hybrid workers’ office and remote workdays, as well as worktimes and non-worktimes. The work environment was modelled as a fixed effect, participant ID was modelled as a random effect to account for repeated observations in the same subject, and sex, age, and BMI were used as covariates to account for between-person differences in the levels of physical activity and SED. The mean 24-h movement behavior compositions (in minutes) for office and remote workdays were calculated based on the predicted values of all ilr coordinates obtained from the previously described linear mixed models. These predicted ilr values were combined and back-transformed to the simplex using the inverse isometric log-ratio (ilr) transformation. For prediction, categorical covariates were fixed at their reference (or most frequent) categories and continuous covariates at their mean values. The resulting compositions were scaled to sum to 1440 min (24 h) or to mean worktime (458 min) or non-worktime (496 min).

As an additional analysis, the potential effect modification by commuting mode on differences in 24-h movement behaviors between office and remote workdays was examined by including an interaction term between work environment (office vs. remote) and commuting mode (passive/active) in the previously described linear mixed models. Moreover, a sensitivity analysis was carried out by including only full measurement days covering the entire 24-h day (sum of sensor wear time during self-reported waking hours and TiB). Inclusion of only full measurement days did not exclude any participants, but led to exclusion of 13 office workdays (6%) and 32 remote workdays (13%).

### Time flow analysis

As a complementary analysis, time flow analysis, described in detail previously [33, 34], was used to illustrate the amount of time flowing between each type of movement behavior between office and remote workdays. Only those participants providing at least two office and two remote workdays, including 24 h of sensor wear time per day, were included in this complementary analysis (*n* = 68, 70% of the total study sample). For each of these participants, two randomly selected office workdays and two randomly selected remote workdays were compared. Sensor data were averaged to a 1-minute level, and time flows were then calculated for each individual by comparing each 1-minute interval on an office workday with the available corresponding 1-minute interval on a remote workday. Given the unmatching worktimes and non-worktimes between the office and remote workdays compared, time flows were only calculated at a daily level. A change in any movement behavior during the 1-minute interval was added to the time flow. Daily time flows were calculated for each individual and averaged across all participants. Results of time flows between movement behaviors (inflows and outflows) were graphically presented in chord diagrams using the *circlize* package in R [35]. Net time flows were further calculated by subtracting the time flows out of a certain movement behavior to another given movement behavior (for instance, from TiB to SED) from the corresponding time flows in (that is, from SED to TiB).

## Results

### Participant characteristics

Participants were on average 42 (SD 10) years old, mostly women (84%), and in professional occupations (85%) (Table 1). Smoking was rare (2%), and most participants reported their health as good (63%) or rather good (28%). There was no difference in sensor wear time during worktime between office and remote work (7 h 38 min for both). However, the sum of sensor wear time during self-reported waking hours and self-reported TiB was shorter on remote workdays when compared to office workdays (23 h 38 min vs. 23 h 51 min).

**Table 1:** Participant characteristics (*n* = 97)

Characteristics	
Age, mean (SD)	42 (10)
Women, *n* (%)	81 (84)
Occupational status, *n* (%)	
Professionals	82 (85)
Associate professionals/service workers	15 (15)
BMI, *n* (%)	
Normal weight*	53 (55)
Overweight	31 (32)
Obese	13 (13)
Current smoking, *n* (%)	2 (2)
Self-reported health**, *n* (%)	
Good	60 (63)
Rather good	27 (28)
Average	9 (9)
Commuting mode, *n* (%)***	
Passive	45 (46)
Active	52 (54)
Valid office workdays, mean (range)	2.4 (1 − 5)
Valid remote workdays, mean (range)	2.6 (1 − 5)
Sum of sensor wear time during waking hours and TiB, mean (SD), min	
Office	1431 (37)
Remote	1418 (53)
Difference (95% CI)	-13 (-26 to -0.1)
Sensor wear time during worktime, mean (SD), min	
Office	458 (63)
Remote	458 (50)
Difference (95% CI)	-0.4 (-15 to 15)

*BMI *body mass index, *TiB *time in bed

*The study population did not include underweight (< 18.5 kg/m2) participants

**The study population did not include any participants reporting their health to be rather poor or poor

***Based on self-reported data

### Differences between office and remote workdays

The model-based compositional means of office and remote workdays are presented in Table 2. On office workdays, participants’ TiB was 7.6 h, they spent 10.6 h sedentary and had 5.0 h LPA and 49 min MVPA per a 24-h day. The ratio of active behaviors (that is, LPA and MVPA) to passive behaviors (that is, TiB and SED) was significantly lower on remote compared with office workdays (*p*<.0001) (Table 3). When expressed in minutes, this corresponded to 14 min longer TiB, 44 min more SED, 37 min less LPA, and 21 min less MVPA, on remote workdays (Table 2). Moreover, the ratio of LPA to MVPA was significantly higher on remote workdays when compared to office workdays (*p*<.0001), reflecting a more pronounced relative difference in MVPA than in LPA (-43% vs. -12% Table 2). The ratio of SED to TiB did not differ between office and remote workdays (*p* = .15).

**Table 2:** The model-based compositional mean hours/minutes for each 24-h movement behavior on office workday, remote workday, and office and remote workday’s worktime and non-worktime during self-reported waking hours, derived from the model adjusted for sex, age, and BMI

	TiB, h (%)	SED, h (%)	LPA, h (%)	MVPA, min (%)
Workday*				
Office	7.6 (32)	10.6 (44)	5.0 (21)	49 (3)
Remote	7.8 (33)	11.3 (47)	4.4 (18)	28 (2)
Difference, min (%)	+ 14 (+ 3)	+ 44 (+ 7)	-37 (-12)	-21 (-43)
Worktime**				
Office	-	5.2 (68)	2.2 (28)	13 (3)
Remote	-	5.9 (76)	1.7 (23)	3 (1)
Difference, min (%)	-	+ 36 (+ 12)	-26 (-20)	-10 (-78)
Non-worktime during waking hours***				
Office	-	5.2 (63)	2.5 (30)	32 (7)
Remote	-	5.5 (67)	2.4 (28)	21 (4)
Difference, min (%)	-	+ 19 (+ 6)	-8 (-5)	-11 (-35)

Due to different scaling, the differences in worktime and non-worktime level do not add up to differences in workday level

*TiB *time in bed, *SED *sedentary time, *LPA *light physical activity, *MVPA *moderate-to-vigorous physical activity

* rescaled to sum up to 1440 min (24 h)

** rescaled to sum up to the mean worktime on workdays (458 min)

*** rescaled to sum up to the mean non-worktime during waking hours on workdays (496 min)

**Table 3:** Estimated fixed effects of differences in the compositional parts and their 95% confidence intervals between office and remote work from the linear mixed models. For workdays, balance coordinate 1 is for active (light physical activity (LPA) and moderate-to-vigorous physical activity (MVPA)) vs. passive movement behaviors (sedentary behavior (SED), time in bed (TiB)), balance coordinate 2 for LPA vs. MVPA, and balance coordinate 3 for SED vs. TiB. For workdays’ worktime and non-worktime, balance coordinate 1 is for SED vs. LPA and MVPA, and balance coordinate 2 for LPA vs. MVPA

	β	95% CI	*P* value
Workday			
Balance coordinate 1: Active vs. passive behaviors			
Office vs. remote	-0.40	-0.51 to -0.28	< 0.0001
Balance coordinate 2: LPA vs. MVPA			
Office vs. remote	0.31	0.19 to 0.42	< 0.0001
Balance coordinate 3: SED vs. TiB			
Office vs. remote	0.03	-0.01 to 0.06	0.15
Worktime			
Balance coordinate 1: SED vs. LPA and MVPA			
Office vs. remote	0.81	0.60 to 1.02	< 0.0001
Balance coordinate 2: LPA vs. MVPA			
Office vs. remote	0.93	0.64 to 1.21	< 0.0001
Non-worktime			
Balance coordinate 1: SED vs. LPA and MVPA			
Office vs. remote	0.25	0.14 to 0.36	< 0.0001
Balance coordinate 2: LPA vs. MVPA			
Office vs. remote	0.27	0.13 to 0.41	0.0003

Adjusted for sex, age, and BMI

*TiB *time in bed, *SED *sedentary time, *LPA *light physical activity, *MVPA *moderate-to-vigorous physical activity

**Fig. 1:**
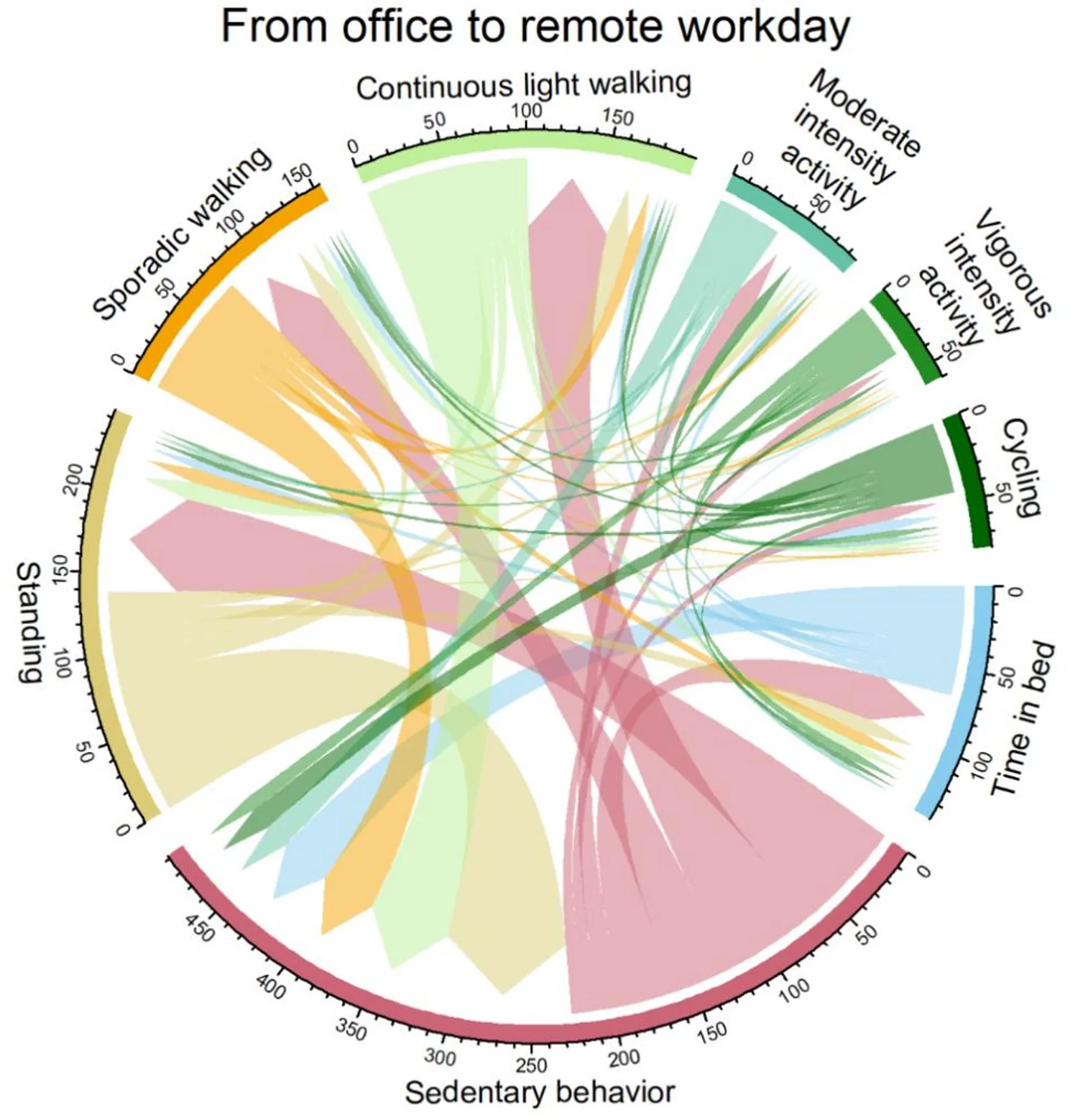
Chord diagram of time flow from office to remote workday between each activity type during the 24-hour day among participants providing at least two office and two remote workdays. For instance, 67 min/day flowed from sedentary behavior to standing, but 86 min/day flowed back from standing to sedentary behavior, leading to a net time flow from standing to SED by 19 min/day

Figure 1 illustrates the time flows, that is, how 24-h movement behaviors replace each other when changing the work environment from office to a remote work environment. The net time flows in minutes per day are given in Supplemental Fig. 1. The main contributor to the higher SED on remote workdays was the net time flow from standing to SED by 19 min/day (Fig. 1, Supplemental Fig. 1). Also, the net time flow from cycling to SED (7 min/day) and from TiB to SED (5 min/day) contributed to the higher SED. Similarly, lower LPA during remote workdays was mainly explained by the net time flow from standing to SED. Lower MVPA was mostly explained by the net time flow from cycling to SED (7 min/day), and from cycling to TiB (4 min/day), while longer TiB was explained by the net time flow from standing and sporadic walking to TiB (8 min/day in total) and from cycling to TiB (4 min/day).

### Differences between office and remote worktime and non-worktime

During office worktime, participants spent 5.2 h in SED, 2.2 h in LPA, and 13 min in MVPA (Table 2). The ratio of SED to the combination of LPA and MVPA was significantly higher during remote worktime compared with office worktime (*p*<.0001) (Table 3), driven by more time spent in SED (36 min), and less time in LPA (26 min), and MVPA (10 min) during remote worktime (Table 2). The ratio of LPA to MVPA was also significantly higher on remote worktime compared to office worktime (*p*<.0001), reflecting a higher relative difference in MVPA than in LPA between office and remote workdays (-78% vs. 20%).

During non-worktime on an office workday, participants spent 5.2 h in SED, 2.5 h in LPA, and 32 min in MVPA. The ratio of SED to the combination of LPA and MVPA was significantly higher on remote workdays’ non-worktime compared to office workdays’ non-worktime (*p*<.0001), driven by more time spent in SED (19 min), and less time spent in LPA (8 min) and MVPA (11 min) during non-worktime on a remote workday (Table 2). The ratio of LPA to MVPA was also higher on remote non-worktime compared to office non-worktime (*p* = .0003).

### Additional analyses

The difference in the ratios between 24-h movement behaviors on remote workdays vs. office workdays did not seem to differ by commuting mode on workdays or during worktime (Supplemental Table 1). However, during non-worktime, the difference in the ratio of SED to the combination of LPA and MVPA tended to be higher among active commuters compared to passive commuters (p for interaction 0.07). When expressed in minutes, this was reflected in substantially larger differences during non-worktime between remote and office workdays among active commuters (SED + 31 min, LPA − 13 min, MVPA − 18 min) than among passive commuters (+ 7 min, -1 min, and − 6 min, respectively; Supplemental Table 2).

To account for the possible effect of sensor wear time difference between office and remote workdays, a sensitivity analysis with only full measurement days with 24 h of sensor wear time was also conducted. The interpretation of the results did not change (Supplemental Table 3).

## Discussion

This study among hybrid workers showed that the proportion of time spent in passive behaviors, that is, TiB and SED, was markedly higher relative to time spent in active behaviors (LPA, MVPA) during remote workdays when compared to office workdays. Time flow analysis indicated that mainly the time spent standing and cycling was replaced with SED and TiB when changing the work environment from office to a remote work environment.

Our study builds on previous findings from the WORKDAY study [17] by showing novel findings on how the composition of all 24-h movement behaviors differs between office and remote workdays and how time spent in different daily behaviors is redistributed when changing the work environment from office to a remote work environment. Our results also update previous device-based evidence, mainly limited to the COVID-19 pandemic period [6]. These previous studies on within-individual differences in wake time/worktime movement behaviors [12–16, 18], TiB/sleep [10, 11], or all 24-h movement behaviors [8, 9] among hybrid workers from Finland, Sweden, Germany, Japan, and Singapore generally showed similar trends of longer TiB, higher SED, and lower physical activity during remote workdays as observed in the current study. However, not all studies found notable differences in SED [9, 15] or LPA [15], which may be due to previous studies’ smaller study samples (*n* = 27–55), and the pandemic context in which several studies were conducted, where contextual conditions could have shaped 24‑h movement behaviors alongside the effects of remote work itself [6, 21].

There are several possible explanations for our findings. Longer TiB on a remote workday, when compared to an office workday, is likely partly attributable to the removal of time required for commuting. This is supported by our finding that the longer TiB was mainly explained by time taken away from standing, sporadic walking, and cycling, and slightly more than half of the study participants reported commuting by walking or cycling. The main differences in 24-h movement behaviors between office and remote workdays were observed in SED during worktime, as standing at the office was replaced with SED at remote work environment. Higher SED during remote worktime may be attributable to not having access to equipment such as a height-adjustable desk at home, the lack of external interruptions, and/or shorter transitions when working from home [36]. Some individuals may choose to schedule work tasks requiring long-term concentration for remote work days, which may result in higher prolonged SED particularly at home [6]. Home environments tend to lack features that commonly serve to break up engagement with long-term work tasks at the office, such as external interruptions, workday structure, and social interactions [36, 37], potentially resulting in longer or more frequent prolonged SED bouts when compared to carrying out similar work tasks at the office. Reduced active commuting may be an important contributor to the lower MVPA observed during remote workdays’ non-worktime. Our additional analyses seemed to support this explanation, as the observed higher proportion of SED relative to physical activity during non-worktime on remote workdays, compared to office workdays, was more pronounced among active than passive commuters. Moreover, time-flow analysis indicated that the lower MVPA on remote workdays was mainly driven by a shift of time spent in cycling (likely accrued from active commuting on office workdays) to SED and TiB.

Regarding the potential health implications of remote workdays, including longer TiB, previous studies have suggested that sleeping longer has the potential to reduce the same day’s perceived stress [38], improve mood (at least among those with usual short sleep) [39], while no such positive effect has been observed for cognitive function [40]. Given that experimental studies have demonstrated acute benefits of reducing SED by interrupting it with physical activity, and to a lesser extent with standing, on postprandial glucose and insulin responses [41, 42], more sedentary and less physically active remote workdays may be associated with less favorable metabolic outcomes. Examination of more detailed accumulation patterns of 24-h movement behaviors was beyond the scope of the current study, but represents an important area for future research, as accumulation patterns of SED and physical activity may influence health outcomes. Overall, further research is needed to examine how remote workdays and the extent of remote working (including fully remote workers) influence health in both short- and long-term, and to determine the optimal balance between office and remote workdays for supporting health, well-being, and work ability.

Our findings can be used to identify potential targets for future intervention studies aiming to promote healthier daily activity patterns in remote work environments, an area where evidence is still limited but growing [43]. Given that remote workdays were markedly more sedentary than office workdays, interventions to reduce SED through physical activity are warranted, especially during remote worktime. As the main shift in movement behaviors from office to remote work appeared to be from standing to SED, height-adjustable desks may represent a potential tool to reduce SED in remote work and they have been shown to be effective in office settings, decreasing worktime SED by up to 70–80 min per day [44, 45]. There is also some preliminary evidence supporting their effectiveness in remote work [46]. However, the provision or acquisition of adjustable desks for remote work might not always be feasible due to resource or space limitations [36, 46]. Furthermore, even if adjustable desks could be provided, it should also be noted that breaking up SED with standing alone may be insufficient to improve cardiometabolic health [41, 47]. Moreover, prolonged and excessive standing may also have unfavorable health consequences, such as musculoskeletal and cardiovascular health risks [48–51]. This highlights the need for strategies that promote not only standing but also physical activity. Another potentially more time-efficient option would be to replace SED with MVPA, as similar health benefits may be achieved with considerably less time than reallocating time to LPA [52]. Promoting MVPA has proven challenging in previous office-based interventions [45], but provisional evidence suggests that increasing MVPA through a multicomponent intervention is feasible and has the potential to increase MVPA on remote workdays [53]. Remote work may offer specific advantages that could be utilized in such interventions, including greater flexibility in arranging working hours and tasks, increased opportunities for meaningful activities, and the absence of some common social barriers to taking breaks from sitting present at the office. These factors may facilitate integrating MVPA into the remote workday, thereby offering new opportunities to increase MVPA [54, 55]. For those who commute long distances by passive modes, time freed from commuting during remote workdays offers an opportunity to increase physical activity. In contrast, those commuting by active modes may need to compensate for the removal of active commuting on remote workdays.

The most important strengths of our study include the within-individual comparison of all 24-h movement behaviors between office and remote workdays, using thigh-worn sensors to measure different postures required for separating movement behaviors, and utilizing the CoDA methodology to take into account the codependency between 24-h movement behaviors. As a limitation, TiB was assessed using self-reported bedtimes and wake times. Therefore, TiB may include periods of sedentary time and/or physical activity, and sleep could not be evaluated or compared between office and remote workdays. Moreover, the generalizability of our results may be limited as we recruited mainly university employees, among whom the level of education and work-related autonomy might be relatively high. In addition, in the present study, we did not examine the potential role of work environment characteristics at home or in the office. Furthermore, detailed information on participants’ work tasks, employer-level policies, health promotion strategies, and participants’ use of such initiatives was not available. Future studies are needed to examine how work environment characteristics, work tasks and demands, and organizational support contribute to differences in movement behaviors between remote and office work.

## Conclusions

Among hybrid workers, remote workdays were characterized by a higher proportion of passive behaviors (TiB, SED), leading to a lower proportion of active behaviors (LPA, including standing, and MVPA) when compared to office workdays. Especially, standing and cycling accumulated during office workdays were replaced with passive behaviors during remote workdays. These findings suggest that exploring feasible ways to compensate for reduced physical activity and increased sedentary behavior during remote work may be warranted, although further research is needed to determine the health implications of differences in 24-h movement behaviors between remote and office workdays.

## Supplementary Information

Supplementary Material 1.

Supplementary Material 2.

## Data Availability

Selected variables and their descriptions without personal identification codes are distributed to investigators and collaborators working on specific projects. The rights to the data belong to the WORKDAY research group. Data sharing outside the WORKDAY group requires a data sharing agreement. Investigators can submit an expression of interest to the WORKDAY research group.

## Abbreviations

BMI: Body mass index
CoDA: Compositional data analysis
ISCO: International Standard classification of Occupations
LPA: Light physical activity
MVPA: Moderate-to-vigorous physical activity
SED: Sedentary behavior
TiB: Time in bed
