# Supplementary material for: How composition of device-measured 24-h movement behaviors differs between office and remote workdays among hybrid workers?
Source: Int J Behav Nutr Phys Act. 2026 Jul 28;23:100. doi: 10.1186/s12966-026-01959-0 (PMC13693173; doi:10.1186/s12966-026-01959-0)
Supplement: Supplementary file 2 [file 12966_2026_1959_MOESM2_ESM.docx]

# **Supplemental material**

# **Supplemental Table 1.** Estimated fixed effects of differences in the compositional parts and their 95% confidence intervals between office and remote workdays from the linear mixed models by commuting mode. For workdays, balance coordinate 1 is for active (light physical activity (LPA) and moderate-to-vigorous physical activity (MVPA)) vs. passive movement behaviors (sedentary behavior (SED), time in bed (TiB)), balance coordinate 2 for LPA vs. MVPA, and balance coordinate 3 for SED vs. TiB. For workdays’ worktime and non-worktime, balance coordinate 1 is for SED vs. LPA and MVPA, and balance coordinate 2 is for LPA vs. MVPA.

|  | β | 95 % CI | P for interaction |
| --- | --- | --- | --- |
| **Workdays** | | | |
| Balance coordinate 1: Active vs. passive behaviors | | | 0.27 |
| Passive commuters | -0.33 | -0.48 to -0.18 |  |
| Active commuters | -0.45 | -0.61 to -0.29 |  |
| Balance coordinate 2: LPA vs. MVPA | | | 0.50 |
| Passive commuters | 0.26 | 0.12 to 0.40 |  |
| Active commuters | 0.34 | 0.16 to 0.53 |  |
| Balance coordinate 3: SED vs. TiB | | | 0.36 |
| Passive commuters | 0.01 | -0.05 to 0.06 |  |
| Active commuters | 0.04 | -0.004 to 0.08 |  |
| **Worktime** | | | |
| Balance coordinate 1: SED vs. LPA and MVPA | | | 0.81 |
| Passive commuters | 0.78 | 0.47 to 1.09 |  |
| Active commuters | 0.83 | 0.54 to 1.12 |  |
| Balance coordinate 2: LPA vs. MVPA | | | 0.96 |
| Passive commuters | 0.92 | 0.52 to 1.32 |  |
| Active commuters | 0.93 | 0.52 to 1.35 |  |
| **Non-worktime** | | | |
| Balance coordinate 1: SED vs. LPA and MVPA | | | 0.07 |
| Passive commuters | 0.14 | -0.01 to 0.29 |  |
| Active commuters | 0.34 | 0.18 to 0.51 |  |
| Balance coordinate 2: LPA vs. MVPA | | | 0.37 |
| Passive commuters | 0.20 | -0.005 to 0.41 |  |
| Active commuters | 0.33 | 0.13 to 0.53 |  |

Notes: Adjusted for sex, age, and BMI. TiB=time in bed, SED=sedentary time, LPA=light physical activity, MVPA=moderate-to-vigorous physical activity

# **Supplemental Table 2.** The model-based compositional mean hours/minutes for each 24-h movement behavior on office workday, remote workday, and office and remote workday’s worktime and non-worktime by commuting mode during waking hours, derived from the model adjusted for sex, age, and BMI.

|  | **Passive commuters** | | | | **Active commuters** | | | |
| --- | --- | --- | --- | --- | --- | --- | --- | --- |
|  | **TiB, h (%)** | **SED, h (%)** | **LPA, h (%)** | **MVPA, min (%)** | **TiB, h (%)** | **SED, h (%)** | **LPA, h (%)** | **MVPA, min (%)** |
| **Workday*** | | | | |  |  |  |  |
| Office | 7.5 (31) | 10.9 (45) | 5.0 (21) | 40 (3) | 7.7 (32) | 10.4 (43) | 4.9 (21) | 58 (4) |
| Remote | 7.7 (32) | 11.4 (47) | 4.5 (19) | 25 (2) | 7.9 (33) | 11.3 (47) | 4.2 (18) | 31 (2) |
| Difference, min (%) | +15 (+3) | +30 (+5) | -30 (-10) | -15 (-38) | +13 (+3) | +56 (+9) | -42 (-14) | -27 (-47) |
| **Worktime**** | | | | |  |  |  |  |
| Office | - | 5.2 (69) | 2.2 (29) | 12 (3) | - | 5.3 (69) | 2.1 (28) | 14 (3) |
| Remote | - | 5.8 (76) | 1.8 (24) | 3 (1) | - | 5.9 (77) | 1.7 (22) | 3 (1) |
| Difference, min (%) | - | +34 (+11) | -24 (-18) | -9 (-78) | - | +39 (+12) | -28 (-21) | -11 (-79) |
| **Non-worktime during waking hours***** | | | | |  |  |  |  |
| Office | - | 5.5 (66) | 2.4 (29) | 24 (5) | - | 5.0 (61) | 2.6 (31) | 42 (8) |
| Remote | - | 5.6 (67) | 2.4 (29) | 18 (4) | - | 5.5 (67) | 2.3 (28) | 24 (5) |
| Difference, min (%) | - | +7 (+2) | -1 (-1) | -6 (-25) | - | +31 (+10) | -13 (-8) | -18 (-43) |

TiB=time in bed, SED=sedentary time, LPA=light physical activity, MVPA=moderate-to-vigorous physical activity. Due to different scaling, the differences in worktime and non-worktime level do not add up to differences in workday level.

* rescaled to sum up to 1440 min (24 h)

** rescaled to sum up to the mean worktime on workdays (458 min)

*** rescaled to sum up to the mean non-worktime during waking hours on workdays (496 min)

# **Supplemental Table 3.** Estimated fixed effects of differences in the compositional parts and their 95% confidence intervals between office and remote work from the linear mixed models from only full measurement days covering the entire 24-h day (sum of sensor wear time during self-reported waking hours and TiB). For workdays, balance coordinate 1 is for active (light physical activity (LPA) and moderate-to-vigorous physical activity (MVPA)) vs. passive movement behaviors (sedentary behavior (SED), time in bed (TiB)), balance coordinate 2 for LPA vs. MVPA, and balance coordinate 3 for SED vs. TiB. For workdays’ worktime and non-worktime, balance coordinate 1 is for SED vs. LPA and MVPA, and balance coordinate 2 is for LPA vs. MVPA.

|  | β | 95 % CI | P value |
| --- | --- | --- | --- |
| **Workday** | | | |
| Balance coordinate 1: Active vs. passive behaviors | | | |
| Office vs. remote | -0.42 | -0.55 to -0.29 | <.0001 |
| Balance coordinate 2: LPA vs. MVPA | | | |
| Office vs. remote | 0.34 | 0.20 to 0.48 | <.0001 |
| Balance coordinate 3: SED vs. TiB | | | |
| Office vs. remote | 0.02 | -0.01 to 0.05 | 0.21 |
| **Worktime** | | | |
| Balance coordinate 1: SED vs. LPA and MVPA | | | |
| Office vs. remote | 0.83 | 0.61 to 1.06 | <.0001 |
| Balance coordinate 2: LPA vs. MVPA | | | |
| Office vs. remote | 1.00 | 0.70 to 1.31 | <.0001 |
| **Non-worktime** | | | |
| Balance coordinate 1: SED vs. LPA and MVPA | | | |
| Office vs. remote | 0.30 | 0.17 to 0.44 | <.0001 |
| Balance coordinate 2: LPA vs. MVPA | | | |
| Office vs. remote | 0.35 | 0.18 to 0.53 | 0.0001 |

Notes: Adjusted for sex, age, and BMI. TiB=time in bed, SED=sedentary time, LPA=light physical activity, MVPA=moderate-to-vigorous physical activity

# **Supplementary Figure 1.** Net time flows from office workday to remote workday between each movement behavior in min day. Red color indicates a net time flow from the behavior, and blue color a net time flow to the behavior.
